# High-pressure minerals in eucrite suggest a small source crater on Vesta

**DOI:** 10.1038/srep26063

**Published:** 2016-05-16

**Authors:** Run-Lian Pang, Ai-Cheng Zhang, Shu-Zhou Wang, Ru-Cheng Wang, Hisayoshi Yurimoto

**Affiliations:** 1 State Key Laboratory for Mineral Deposits Research, School of Earth Sciences and Engineering, Nanjing University, Nanjing 210046, China; 2 Department of Natural History Sciences, Hokkaido University, Sapporo 060-0810, Japan

## Abstract

High-pressure minerals in meteorites are important records of shock events that have affected the surfaces of planets and asteroids. A widespread distribution of impact craters has been observed on the Vestan surface. However, very few high-pressure minerals have been discovered in Howardite-Eucrite-Diogenite (HED) meteorites. Here we present the first evidence of tissintite, vacancy-rich clinopyroxene, and super-silicic garnet in the eucrite Northwest Africa (NWA) 8003. Combined with coesite and stishovite, the presence of these high-pressure minerals and their chemical compositions reveal that solidification of melt veins in NWA 8003 began at a pressure of >~10 GPa and ceased when the pressure dropped to <~8.5 GPa. The shock temperature in the melt veins exceeded 1900 °C. Simulation results show that shock events that create impact craters of ~3 km in diameter (subject to a factor of 2 uncertainty) are associated with sufficiently high pressures to account for the occurrence of the high-pressure minerals observed in NWA 8003. This indicates that HED meteorites containing similar high-pressure minerals should be observed more frequently than previously thought.

Hyper-velocity collisions between celestial bodies create impact craters on the surfaces of planets and asteroids and may result in the formation of high-pressure minerals^1^,^2^. Many high-pressure minerals have been observed in shocked meteorites^3^,^4^,^5^,^6^,^7^, samples returned by the Apollo missions^8^, and in terrestrial impact structures^9^, indicating that widespread and intense dynamic events have occurred on both planets and asteroids. These minerals provide important clues that allow us to constrain the prevailing Pressure-Temperature-time (*P-T-t*) conditions characterizing these shock events, the sizes and velocities of the impactors, and the size scale of the source impact craters^3^,^4^,^5^,^6^,^7^,^10^,^11^,^12^,^13^. Recently, the *DAWN* mission revealed that impact craters are abundant on the surface of the asteroid 4 Vesta (herein referred to as ‘Vesta’), including two giant impact basins known as Rheasilvia and Veneneia^14^,^15^. It has been proposed that meteorites of the Howardite-Eucrite-Diogenite (HED) clan might be originated during the formation of these two impact basins on Vesta^15^,^16^. In contrast to the widespread impact craters on the Vestan surface, very few high-pressure minerals have been discovered in HED meteorites, possibly due to the formation/generation mechanism of meteorites from their parent bodies^17^. For example, low-level impact events that occur via spallation can result in high ejection velocities of meteorites with few or no high-pressure minerals^17^. However, the occurrence of spallation that launched meteorites from their parent bodies cannot account for the extreme rarity of high-pressure minerals in HED meteorites. Approximately 1,700 HED meteorites have been identified in the meteorite collections; however, high-pressure minerals have only been reported in one shocked eucrite^18^. It remains puzzling that high-pressure minerals are extremely rare in HED meteorites compared with their frequency in Martian and lunar meteorites^19^,^20^,^21^,^22^. To solve this mystery, we performed a survey of high-pressure minerals in shocked HED meteorites. The high-pressure minerals tissintite, vacancy-rich clinopyroxene, and super-silicic garnet are observed in the shocked eucrite Northwest Africa (NWA) 8003 in addition to coesite and stishovite. The discovery of these high-pressure minerals provides important constraints on the *P-T-t* conditions of shock metamorphism in HED meteorites. Given these conditions, we further estimate the possible sizes of the impact craters from which HED meteorites with high-pressure minerals might have originated.

## Results

### Petrology of the host rock and shock melt veins

NWA 8003 is a basaltic eucrite that consists mainly of pyroxene and plagioclase with a subophitic texture. Minor minerals include quartz, troilite, chromite, ilmenite, Ca-phosphate (apatite and merrillite), and zircon. Iron-nickel (FeNi) metal grains are also observed in the fragment of NWA 8003 under consideration (Supplementary Fig. 1). These grains might have an exotic origin^23^,^24^. Most of the pyroxene grains in the host rock show augite exsolution lamellae (usually 1–5 μm wide) within Fe-rich orthopyroxene (Supplementary Fig. 2). The average compositions (Supplementary Table 1) of orthopyroxene (En_33.2±0.5_Fs_64.5±0.6_Wo_2.3±0.5_; based on 12 analyses) and augite (En_27.6±0.3_Fs_28.6±0.3_Wo_43.7±0.4_; 14 analyses) suggest an equilibrium temperature of 718 °C (ref. 25). Most of the plagioclase grains (An_84–92_; Supplementary Table 2) in the host rock are lath-shaped, exhibiting numerous irregular cracks (Supplementary Fig. 2). Most of the plagioclase grains adjacent to melt veins are fully transformed into maskelynite, whereas those farther from the melt veins are usually partially transformed into maskelynite.

**Figure 1:**
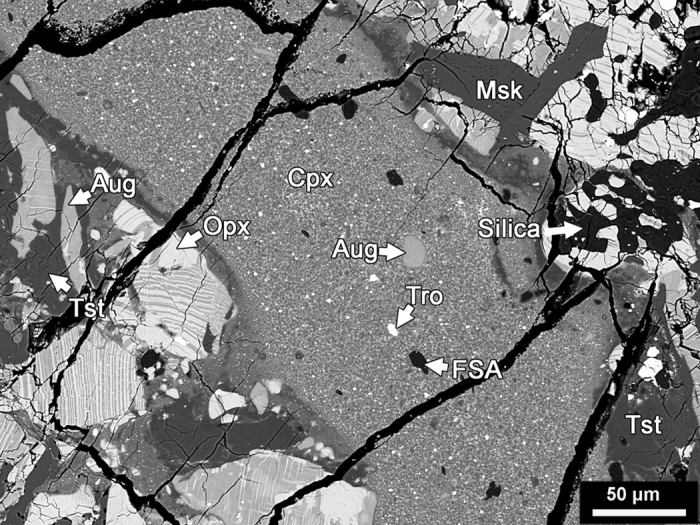
Back-scattered electron image of a typical, thin shock melt vein in NWA 8003. It consists dominantly of clinopyroxene with minor mineral fragments and fine-grained silica aggregates. Cpx: clinopyroxene; Opx: orthopyroxene; Aug: augite; Tro: troilite; Msk: maskelynite; Tst: tissintite; FSA: fine-grained silica aggregate. This figure is generated with BSE images by using Adobe Photoshop^®^ CS2.

**Figure 2:**
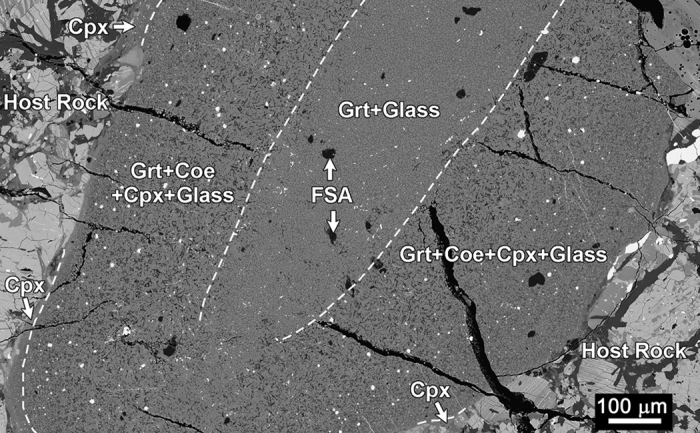
Back-scattered electron image of a typically zoned shock melt vein in NWA 8003. Cpx: clinopyroxene; Grt: garnet; Coe: coesite; Cpx: clinopyroxene; FSA: fine-grained silica aggregate. Dashed lines show the boundaries between different zones of the melt vein. This figure is generated with BSE images by using Adobe Photoshop^®^ CS2 and Illustrator^®^ CS4.

**Figure 3:**
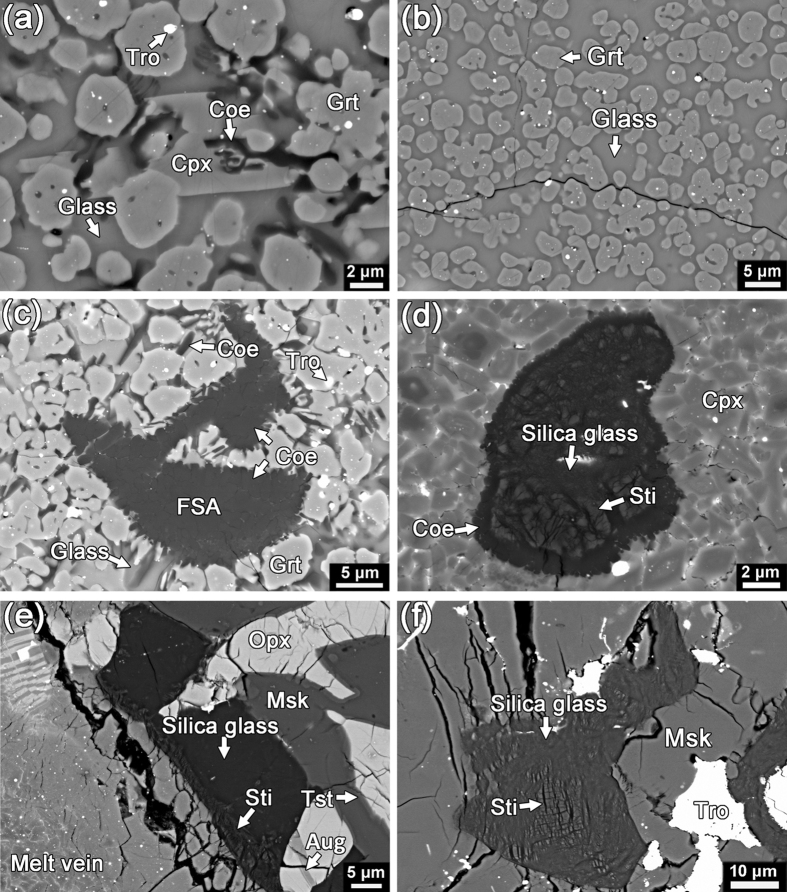
Back-scattered electron images of high-pressure minerals in NWA 8003. (**a**) A typical area showing the textural association of garnet, rod-like coesite, and clinopyroxene embedded in glass. (**b**) Garnet + glass in the central zone of a zoned melt vein. (**c**) Fine-grained silica aggregates dominated by coesite. (**d**) A FSA within a melt vein composed of fine-grained clinopyroxene. Granular coesite forms the rim of the aggregate while stishovite and silica glass occur at the core. (**e**) A quartz grain adjacent to a melt vein has transformed to stishovite and silica glass. Fine-grained lamellae-like stishovite occurs at the boundary with the melt vein. (**f**) Lamellae-like stishovite embedded in silica glass which is close to but not directly contact with a melt vein. Tro: troilite; Coe: coesite; Cpx: clinopyroxene; Grt: garnet; FSA: fine-grained silica aggregate; Sti: stishovite; Msk: maskelynite; Opx: orthopyroxene; Aug: augite. This figure is generated with BSE images by using Adobe Photoshop^®^ CS2.

Shock melt veins are common in NWA 8003 (Supplementary Fig. 1; bulk compositions of representative melt veins are provided in Supplementary Table 3). Their widths vary from a few micrometers to ~1 mm. Most of the melt veins in NWA 8003 are relatively thin (<200 μm wide) and consist predominantly of fine-grained clinopyroxene (<3 μm in size; Fig. 1). A few wide melt veins in NWA 8003 exhibit complex zoning in their mineral assemblage (Fig. 2). They may consist of an edge zone (widths ranging from a few micrometers to ~100 μm in width) dominated by fine-grained clinopyroxene (Fig. 2), a middle zone composed of eclogitic mineral assemblage (garnet + rod-like coesite + clinopyroxene ± glass; Fig. 3a), and a central zone of garnet + glass (Fig. 3b). The texture and mineralogy of the clinopyroxene-dominant edge zone in zoned melt veins are almost identical to those in the thin melt veins (Fig. 1). The eclogitic mineral assemblage in NWA 8003 varies in grain size and mineral abundance among veins and even within a given melt vein. Glass is also present within the zone dominated by the eclogitic mineral assemblage close to the garnet + glass zone (Fig. 3a). The garnet + glass zone is present only in a few wide veins (>~500 μm wide).

In addition to relict fragments of host rock, fine-grained silica aggregates (FSAs), which consist of coesite, stishovite and amorphous silica, are observed within most melt veins (Figs 1, 2, and 3c–d). In contrast to the rod-like coesite within the eclogitic mineral assemblage (Fig. 3a), the coesite in FSAs usually appears as polycrystalline aggregates (Fig. 3c). In a few large FSAs, coesite grains form a thin rim on a large core dominated by lamellae-like stishovite and silica glass (Fig. 3d). Quartz grains adjacent to melt veins are transformed to its high-pressure polymorphs (stishovite and coesite) and/or silica glass (Fig. 3e–f), where stishovite also appears lamellae-like in texture (Fig. 3e–f). However, the textures of quartz and its high-pressure polymorphs (Fig. 3e–f) adjacent to melt veins are different from those of FSAs (Fig. 3c–d) within the melt veins. The former is usually irregular in shape whereas the latter often has a curved outline. Silica phases in the former are rarely centrally zoned, whereas such zoning is commonly observed in FSAs (Fig. 3d).

**Figure 4:**
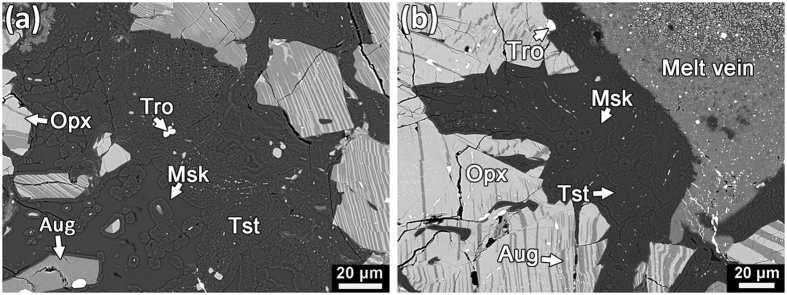
Back-scattered electron images of tissintite occurs in maskelynite adjacent to shock-melt veins. Most of them grow on the mineral fragments in the maskelynite. Tro: troilite; Tst: tissintite; Msk: maskelynite; opx: orthopyroxene; aug: augite. This figure was generated with BSE images by using Adobe Photoshop^®^ CS2.

Tissintite, a high-pressure polymorph of Ca-rich plagioclase with a clinopyroxene structure, is commonly observed in maskelynite adjacent to melt veins. It appears to have nucleated and grown on relict mineral fragments in maskelynite (Fig. 4a–b). Most aggregates of tissintite are small in size (~5 μm), with a few reaching up to ~20 μm.

### Characterization of high-pressure minerals

Tissintite in NWA 8003 is identified by its Raman spectrum and its Electron Back Scatter Diffraction (EBSD) pattern. The Raman spectrum of tissintite is distinctly different from that of anorthite in NWA 8003; instead, it is similar to that of jadeite instead (Supplementary Fig. 3). Its EBSD pattern can be well indexed with the *C2/c* clinopyroxene structure (Supplementary Fig. 4). Chemically, NWA 8003 tissintite is indistinguishable from plagioclase and maskelynite in NWA 8003 (Supplementary Table 2). Based on six oxygen atoms, the tissintite in NWA 8003 has an average chemical formula (Ca_0.64_Na_0.09_□_0.27_)(Al_0.99_Fe_0.01_)(Si_1.62_Al_0.38_)O_6_ with high vacancy concentrations on the M2 site.

The structure of clinopyroxene in the shocked melt veins of NWA 8003 is confirmed by its Raman spectrum and EBSD pattern (Supplementary Figs 5 and 6). Clinopyroxene in both thin and wide melt veins is characterized by a complex chemical composition, with much higher Al_2_O_3_ concentration and more abundant Ca-Eskola (Ca-Esk, Ca_0.5_□_0.5_AlSi_2_O_6_) components than high-Ca and low-Ca pyroxenes in the host rock of NWA 8003 (Supplementary Table 1). Clinopyroxene, when located within the eclogitic mineral assemblage of zoned veins contains slightly higher Na_2_O and CaO than those in either thin melt veins or the edge zones of zoned melt veins. The calculated abundance of the Ca-Esk components (41 ± 8 mol% on average; based on 13 analyses) in the former is generally higher than that in the latter (34 ± 7 mol% on average; 19 analyses).

Garnet occurs as fine dendritic or anhedral to euhedral grains in the zoned melt veins of NWA 8003 (Fig. 3a–c). Its structure is confirmed by its Raman spectrum and EBSD pattern (Supplementary Figs 5 and 7). Our Electron Probe Micro-Analyzer (EPMA) results (Supplementary Table 4) reveal that all garnets are super-silicic, containing 6–13 mol% majorite components [Mg_3_(Mg^VI^Si)^IV^Si_3_O_12_]. The garnet grains within the eclogitic mineral assemblage contain slightly higher Si concentrations (3.09–3.13 atoms per formula unit, apfu; based on 12 analyses) than those in the central zone (3.06–3.09 apfu, 9 analyses), based on 12 oxygen atoms. In contrast, the Al concentrations in the former (1.70–1.80 apfu) are slightly lower than those in the latter (1.77–1.83 apfu).

Stishovite and coesite in NWA 8003 are identified on the basis of their Raman spectra (Supplementary Fig. 8) and EBSD patterns. Although silica glass is also present, as inferred from the petrographic texture, Raman spectra, and EBSD patterns, no other high-pressure polymorphs (e.g., seifertite) were identified.

## Discussion

This study has identified five high-pressure minerals (tissintite, vacancy-rich clinopyroxene, super-silicic garnet, coesite and stishovite). Among these, tissintite, vacancy-rich clinopyroxene, and super-silicic garnet are reported in HED meteorites for the first time. Additionally, this is the first time that tissintite has been observed in samples other than those from shocked Martian meteorites^26^,^27^,^28^. Its presence in NWA 8003 confirms the prediction that tissintite could be common in shocked samples containing calcic plagioclase^14^. Compared with the type tissintite from the Tissint Martian meteorite, the tissintite in NWA 8003 is more calcic, similar to that of Na-free end-member (Ca_0.75_□_0.25_)Al(Si_1.5_Al_0.5_)O_6_ (ref. 27). This composition is related to the originally Ca-rich properties of plagioclase precursors in eucrite. Although tissintite has an identical compositions to plagioclase and maskelynite in NWA 8003, almost all of the tissintite grains appear as intergrowths on tiny pyroxene grains in maskelynite or on rims of large pyroxene grains. This indicates that the tissintite in NWA 8003 formed through direct crystallization from maskelynite, which is similar to the formation mechanism in Martian meteorites^27^.

Differing from orthopyroxene and augite in the host rock, the fine-grained clinopyroxene in the melt veins is unique, containing high concentrations of Al and cation vacancies. Since pyroxene, characterized by abundant cation vacancies, is only observed udner high-pressure conditions^27^,^29^,^30^, the presence of this chemical property indicates that vacancy-rich clinopyroxene in the melt veins formed through rapid crystallization from high-pressure melts. This view is also supported by the texture of the thin melt veins and the edge zones of zoned melt veins. Super-silicic garnet and its associated clinopyroxene and rod-like coesite in NWA 8003 have a fine-grained, igneous texture, which indicates that they also crystallized rapidly from high-pressure melts.

The lamellae-like texture of stishovite in NWA 8003 implies a structure-controlled phase transformation, which is consistent with a solid-state phase transformation mechanism. If stishovite formed through rapid crystallization from high-pressure silica glass, it would be randomly orientated because of rapid nucleation. Therefore, it is very likely that the lamellae-like stishovite in NWA 8003 was also formed through a solid-state phase transformation^18^. Previous studies have reported that seifertite in some Martian meteorites has a similar lamellae-like texture^31^,^32^, possibly indicating a seifertite-related origin for stishovite. However, previous experiments have revealed that although seifertite can form at a low pressure down to 11 GPa, at a temperature of 640–860 K from α-cristobalite^33^, it will transform to stishovite above this temperature range. In addition, for a start material of quartz, much higher pressures (>25 GPa at 900 K) are required to produce seifertite in their experiments. Instead, stishovite forms at a much lower pressure (18 GPa at 800 K). Given that quartz occurs as the low-pressure silica phase in NWA 8003, stishovite might have formed through direct transformation from quartz. If seifertite represents a metastable phase before the formation of stishovite, an anomalously high pressure (probably >25 GPa) would be required to produce seifertite, compared with the pressure inferred from other pressure indicators, as discussed below. To be conservative, we suggested that the stishovite is a product of a solid-state phase transformation from quartz and is not seifertite-related in origin.

In some FSAs, coesite has a polycrystalline texture (Fig. 3c), indicating that the granular coesite crystallized from high-pressure silica glass. Meanwhile, as opposed to the solid-state phase transformation from quartz to coesite which is sluggish owing to a large kinetic barrier^34^, crystallization of coesite from high-pressure silica glass can reduce the kinetic barrier^18^. This makes it plausible that coesite completes its crystallization in a relatively short time. The centrally zoned texture of some FSAs can be accounted for by the presence of a temperature gradient between a silica glass rim and a relict solid portion. During shock-induced melting, the rim of FSAs might have been heated to the melting temperature of quartz; however, the core was not heated sufficiently to reach its melting temperature. Based on the phase diagram for silica, at a given high pressure, the hot rim of quartz will transform into coesite, whereas the relatively cold core will transform into stishovite^35^,^36^. Amorphous silica was originated later from stishovite during adiabatic decompression, since stishovite is sensitive to heating^18^. This interpretation is consistent with the unique occurrence of FSAs in melt veins, whose formation indicates much higher temperatures than those inferred from the host rock^4^. Because zoned FSAs are enclosed by melt veins, it would have been easy to form a hot rim in quartz. However, it would have been difficult to form such a hot rim for quartz grains in the host rock.

Several indicators could potentially be used to constrain the prevailing shock conditions affecting NWA 8003, particularly the stability fields for (1) tissintite, (2) vacancy-rich clinopyroxene, (3) high-pressure polymorphs of silica (coesite and stishovite), (4) the eclogitic mineral assemblage garnet + clinopyroxene + coesite, and (5) the Si and Al concentrations of super-silicic garnet within the eclogitic mineral assemblage. There is no doubt that the presence of tissintite and vacancy-rich clinopyroxene indicates high-pressure conditions. However, their presence does not yield precise constraints on the prevailing shock pressure. (i) Because tissintite is absent in the phase diagram of plagioclase, it should be a metastable phase rather than an equilibrium phase^27^. Although it has been observed in shocked Martian meteorites that were possibly shocked up to >14 GPa^26^,^28^, the exact stability field of tissintite remains unknown. Therefore, it is difficult to constrain the formation pressure based on the presence of tissintite alone. (ii) There are two reasons to suggest that the clinopyroxene in melt veins, characterized by a complex composition, is not a good indicator of pressure. First, clinopyroxene has a wide stability field (from <2 GPa to >20 GPa; ref. 37); hence, it is difficult to constrain the shock pressure based only on the presence of clinopyroxene. Second, the Ca-Esk component in clinopyroxene is a function of both pressure and temperature^30^. With temperature unknown, pressure cannot be constrained precisely, and *vice versa*.

By adopting the static high-pressure phase diagram for pure silica, it has been estimated^18^ that the shock pressures required for the coexistence of coesite and stishovite in Béréba could range from ~8 to ~13 GPa. However, in relation to the FSAs in NWA 8003, stishovite and coesite have formed through different transformation mechanisms (i.e., solid-state phase transformation for stishovite and crystallization from high-pressure silica glass for coesite). Although a shock pulse could last for several tens of milliseconds, kinetic effects might be non-negligible when applying static high-pressure phase diagrams to interpret solid-state phase transformations of minerals. For instance, recent shock compression experiments reveal that although stishovite might form within 1.4 ns, the nucleation pressure exceeds 18 GPa (ref. 38), which is much higher than the transformation pressure from coesite to stishovite (~8 GPa) under static high-pressure experiments^39^. Therefore, large uncertainties might affect the resulting shock-pressure conditions based on the coexistence of coesite and stishovite, which might have formed through different mechanisms. Without being able to quantitatively constrain the kinetic effect associated with the formation of coesite and stishovite, the coexistence of coesite and stishovite in NWA 8003 only indicates that the shock pressure should be >~8 GPa, which is the lower pressure limit required for the presence of stable stishovite^39^.

Compared with the first three indicators described above, the latter two (i.e., the eclogitic mineral assemblage garnet + clinopyroxene + coesite, and the Si and Al concentrations of super-silicic garnet within the eclogitic mineral assemblage) might provide better constrains on the shock conditions. Static high-pressure phase diagrams can be applied to this eclogitic mineral assemblage, because the eclogitic mineral assemblage of garnet, clinopyroxene, and rod-like coesite crystallized from the high-pressure melt veins in NWA 8003. Based on the static high-pressure experiments aimed at studying anhydrous mid-ocean ridge basalt^40^, an eclogitic mineral assemblage containing glass indicates a pressure between 5 GPa and 10 GPa. If the pressure exceeds 10 GPa, stishovite would be stable instead of coesite and would coexist with garnet and clinopyroxene. As discussed above, the stishovite in NWA 8003 formed through a solid-state phase transformation, and it is not in equilibrium with garnet or clinopyroxene, which crystallized from melts. Consequently, the eclogitic mineral assemblage including glass in the melt veins indicates that the eclogitic mineral assemblage crystallized at a pressure ranging from ~5 GPa to ~10 GPa.

**Figure 5:**
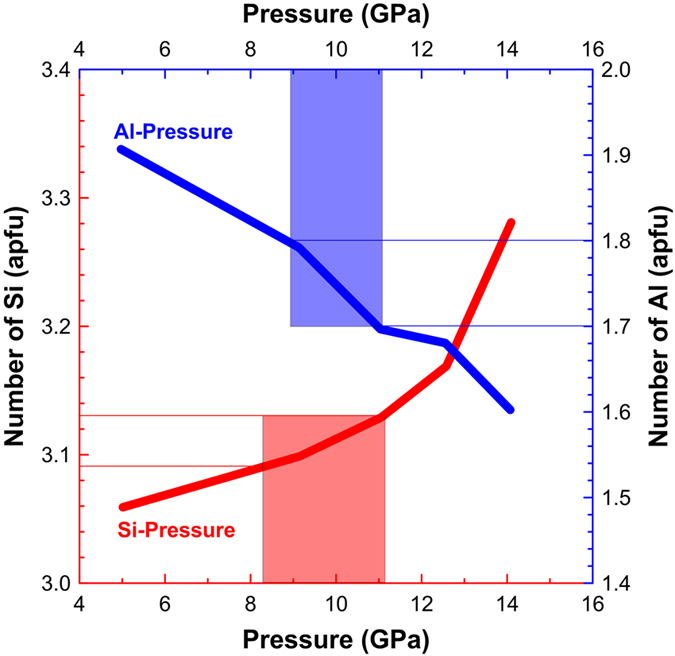
Correlation diagram for atomic numbers of Si and Al of garnet within the eclogitic mineral assemblage *versus* pressure (modified from ref. 41). The inferred pressure ranges from the Si concentrations of garnet (illustrated by the red rectangular region) are generally consistent with those from the Al concentrations (illustrated by the blue rectangular regions).

Static high-pressure experiments on natural mid-oceanic ridge basalt reveal that the Si and Al concentrations in super-silicic garnet are a function of pressure^41^. Since the super-silicic garnet grains in NWA 8003 crystallized from high-pressure melt, the results of static high-pressure experiments are used to estimate the pressure under which super-silicic garnet with a specific composition may have formed. Therefore, based on a diagram of atomic numbers of Si and Al (based on 12 oxygen atoms) versus pressure (Fig. 5), the compositions of garnet within the eclogitic mineral assemblage correspond to pressures of ~8.5 to ~10 GPa. The pressure inferred from the composition of garnet within the eclogitic mineral assemblage is well consistent with that inferred from the stability field of the eclogitic mineral assemblage (~5 to ~10 GPa), and has a better precision.

Since shock melt veins cool predominantly by conduction to the surrounding host rock, crystallization of zoned melt veins will proceed from the edge to the centre^4^. Thus, zones that crystallized early may record higher pressures than those that crystallized later. This implies that the pressure during crystallization of the clinopyroxene-dominant melt-vein zone might be higher than that representative of the zone dominated by the eclogitic mineral assemblage, which is in turn higher than that for the central zone composed of garnet and glass. Therefore, the pressure during the crystallization of clinopyroxene-dominant zones is of the order of, or higher than, 10 GPa, and the pressure during crystallization of the garnet + glass zone was possibly less than ~8.5 GPa. With reference to the arguments put forward above, a rough pathway for the shock metamorphism of NWA 8003 can be deduced. During the early stage of shock decompression, shock melt veins and maskelynite formed, quartz grains within the melt veins transformed to fine-grained aggregates composed of coesite, stishovite, and amorphous silica, and plagioclase transformed to maskelynite. Subsequently, tissintite crystallized from maskelynite, and shock melt veins started to crystallize. During crystallization of the clinopyroxene-dominant veins and clinopyroxene-dominant zones within zoned melt veins, the pressure might have been ~10 GPa or higher. When the pressure dropped to ~8.5–10 GPa, the eclogitic mineral assemblage started to crystallize. Next, the garnet + glass zone solidified, probably at a pressure of <~8.5 GPa. If we assume a shock pressure of ~10 GPa, the melting temperature of such a basaltic material similar to that found in NWA 8003 should be >1900 °C based on the phase diagram for mid-oceanic ridge basalt^40^.

The presence of super-silicic garnet in the central zone of some veins indicates that melt veins were under high pressure when solidification finished. Based on the thermal conduction model developed in the literature^10^,^13^,^42^, the lower limit to the shock duration can be estimated from the solidification time of the melt veins (see Methods section). Given that the melt veins in NWA 8003 began to solidify at a pressure of 10 GPa and that the temperatures of the host rock and melt veins during the shock compression were 100 °C and 2000 °C, respectively, the calculated solidification time is ~70 ms for a melt vein of 1 mm wide. This indicates that the shock pulse lasted at least ~70 ms on scale. Although the initial temperature difference between the host rock and melt veins was not exactly 1900 °C, previous studies have shown that different initial temperature differences (up to 1000 °C) does not change the scale of the shock pulse^10^. Instead, the solidification time depends mainly on the width of the melt veins^10^.

**Figure 6:**
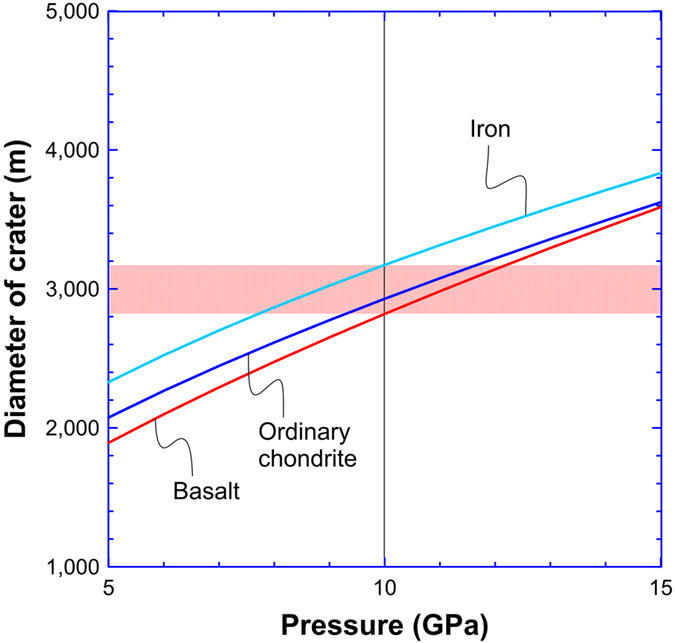
Diameter of crater as a function of shock pressure assuming a basaltic target and a shock pulse of 72 ms. Different curves denote impactors of different materials. When shock pressure is 10 GPa and shock duration is 72 ms, the size of crater is 2.8–3.2 km for impactors with various densities.

By applying simulation models developed in previous studies^1^,^2^ (see Methods section), we can calculate the impact velocity and size of the impactor that produced the shock melt veins in NWA 8003, as well as a lower limit to the diameter of the source crater. If we assume impactors spanning a range of densities (basalt, ordinary chondrite, and iron), an impactor of 0.16–0.18 km in size is required to produce an impact pressure of 10 GPa, vertically impacting the basaltic surface of Vesta with an impact velocity of 1.2–1.7 km s^−1^. The corresponding diameter of the transient impact crater would be 2.8–3.2 km (Fig. 6). Note that these calculation results obtained here represent their lower limits for the impact velocity, impactor size, and crater size, respectively. For instance, the impactor might not impact the surface vertically. In that case, a higher impact velocity would be required to account for the vertical velocity adopted in our study. If we consider a possible pressure gradient within a large impact crater, the impact crater might be much larger than ~3 km. However, the impact velocity (~1.5 km s^−1^) that is required to account for the formation of the high-pressure mineral assemblages in NWA 8003 is consistent on magnitude with the average impact velocity (~5 km s^−1^) at the location of Vesta in the main asteroid belt^43^,^44^. This indicates that high-pressure minerals that formed under similar shock pressures might be more common than previously thought, assuming that HED meteorites indeed originated from the asteroid 4 Vesta. Additional investigations of the shock-melt veins in HED meteorites are needed to confirm this prediction. In addition, considering the uncertainty of a factor of 2 (refs 1,45), the diameter of the transient impact crater required to account for the high-pressure mineral assemblages in NWA 8003 is also comparable to the sizes of most impact craters (>4 km) on the surface of Vesta^14^. This result indicates that HED meteorites with similar high-pressure mineral assemblages might not necessarely originate from the two giant impact basins on Vesta, as proposed in the literature^15^. They probably formed in smaller impact craters on the surface of Vesta.

## Methods

### Petrographic observations

The petrographic texture of NWA 8003 was observed using a JEOL 7000F field emission gun scanning electron microscope (FEG-SEM) at Hokkaido University, Japan. The accelerating voltage and electron beam current were 15 kV and 3–5 nA, respectively. The mineral chemistry was determined using a JEOL 8100 Electron Probe Micro-Analyzer (EPMA) with wavelength dispersive spectrometers (WDS) at Nanjing University, China. Measurements of most minerals were performed with a focused beam of 20 nA, accelerated at 15 kV, whereas measurements of plagioclase and its polymorphs were performed with a defocused beam (2–5 μm in diameter) at the same beam current and accelerating voltage. Natural and synthetic standards were used. Typical detection limits for oxides of most elements were better than 0.02 wt%. All EPMA data were reduced using the ZAF correction procedure. The bulk compositions of melt veins in NWA 8003 were obtained by using the analytical mode of outlined materials of the PointID Navigator in the Oxford EDS system (INCA software) attached to a JEOL 6490 scanning electron microscope at Nanjing University.

### Raman and electron backscatter diffraction patterns

Characterization of the crystal structures of minerals in NWA 8003 was performed by obtaining their Raman spectra and Electron Backscatter Diffraction (EBSD) patterns, and comparing these with the spectra and patterns in datasets. Raman spectra were measured with the laser micro-Raman spectrometer Renishaw RM2000 at Nanjing University. A microscope was used to focus the excitation laser beam of 514 nm (Ar^+^ laser) on the target phases. The sample was excited by a laser power of ~5 mW, using a spot size of ~2–3 μm. The Raman spectra baselines were reduced by using the OPUS software (version 3.1). The minerals’ EBSD patterns were obtained using an Oxford EBSD detector attached to a JEOL 7000F FEG-SEM at Hokkaido University, at an accelerating voltage of 20 kV and an incident beam current of 4 nA. Before analysis, the sample was vibro-polished and carbon-coated. The experimental EBSD patterns were indexed using the Aztec software with structures from both the HKL dataset and a dataset from the Mineralogical Society of America. The Aztec software automatically suggests indexing solutions ranked by the lowest ‘mean angular deviation’ (MAD) as a ‘goodness of fit’. MAD values of <1 are considered desirable for accurate solutions.

### Estimating the solidification time of shock melt veins

Based on the model developed by Turcotte and coauthors^42^,^46^, the solidification of magmas can be treated as the Stefan Problem involving phase changes (e.g., from liquid to solid) and the latent heat of crystallization. This model has been used to constrain the solidification time of melt veins in shocked meteorites^10^,^13^. We used this model to estimate the solidification time of melt veins in NWA 8003. In this model, molten veins are viewed as a thin slab of width *2w*, bound by two semi-infinite spaces. The solidification time *t*_*s*_ can be determined through


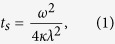


where ω is the half-width of the slab, *κ* is the thermal diffusivity of the molten slab and surrounding material and *λ* is a dimensionless coefficient. The *λ* value can be obtained by solving the transcendental equation,


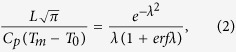


where *L* is the crystallization latent heat, *C*_*p*_ is the specific heat at constant pressure, *erf* is the error function, *T*_*m*_ is the melting temperature of rocks, and *T*_0_ is the initial temperature of the surrounding material.

In addition, we can obtain the temperature at the boundary between the slab and surrounding material, *T*_*b*_, when the melt veins are completely solidified:


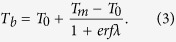


For garnet-bearing shock melt veins in NWA 8003, *T*_*m*_ is assumed to be 2000 °C based on the liquidus temperature of an anhydrous mid-ocean ridge basalt at ~10 GPa^40^. The initial temperature *T*_0_ is the sum of the pre-shock initial temperature and temperature increase induced by the shock. The pre-shock temperature of basaltic rocks under a pressure of ~20–22 GPa is approximately 50–100 °C^47^. Since the solidification time is largely insensitive to the temperature difference (*T*_*m*_ − *T*_0_) compared with the width of melt veins^10^, we adopted T_0_ = 100 °C. The apparent widths of the shock-melt veins in NWA 8003 are up to 1 mm; i.e. *w* = 0.5 × 10^−3^ m. Given the typical values of *L* = 320 kJ kg^−1^, *C*_*p*_ = 1.2 kJ kg^−1^, *κ* = 10^−6^ m^2^ s^−1^ (ref. 42), we obtained that *λ* = 0.93, *t*_*s*_ = 72 ms, and *T*_*b*_ = 1150 °C. These results show that when the vein completely solidifies, the temperature within the vein is still high. It is therefore possible that stishovite transformed into the amorphous phase after heating during shock decompression.

### Estimating the Impact velocity (*v*_*i*_)

A rough estimate of the particle velocity, *u*, and the impact velocity, *v*_*i*_, can be obtained from the planar impact approximation and use of the Hugoniot equation^1^,^2^. The Hugoniot equation, which is based on the conservation of momentum, is:


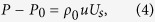


where *P* is the shock pressure, *P*_*0*_ is the pressure before the impact, *ρ*_*0*_ is the density of the material before the impact, *u* is the particle velocity and *U*_*s*_ is the shock wave velocity.

A linear relation between the shock wave and particle velocities can be specified by


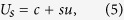


where *c* and *s* are empirical constants whose values depend on the specific materials targeted. Substituting Equation (5) into Equation (4), we have


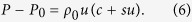


Given the shock pressure *P* and the density (*ρ*_*0*_) of the pre-shock material, the particle velocity *u* of the compressed material (either the target or the projectile) can be obtained by solving Equation (6).

If the target and the projectile are made of the same material, relation between the particle and impact velocities^46^ is


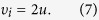


In the case of NWA 8003, we adopt a shock pressure *P* = 10 GPa and *P*_*0*_ = 0 GPa, as well as *ρ*_*0*_ = 2.860 g cm^−3^, c = 2.6 km s^−1^ and s = 1.62 for basalt^1^. Substituting these values into Equations (5)–(7), 6, 7, we obtained *u*_(basalt)_ = 0.87 km s^−1^, *v*_*i*(basalt)_ = 1.74 km s^−1^, and *U*_*s*(basalt)_ = 4.01 km s^−1^.

If the target and the projectile have different densities, the impact velocity can be obtained by solving these equations using the planar impact approximation. The relations between the particle and impact velocities are expressed as


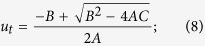



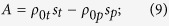



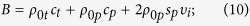



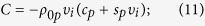



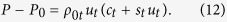


Equation (11) is, in fact, a combination of Equations (4) and (5), and the subscripts ‘*p’* and ‘*t’* refer to the projectile and target, respectively. Here, the target is basalt or at least mostly basalt. Although minor FeNi metal grains are present, no regolith materials (e.g., impact melt glass beads or agglutinates) are observed in NWA 8003. Since large FeNi metal grains are observed in NWA 8003 (Supplementary Fig. 1), iron or ordinary chondrite (OC) projectiles are considered as the projectile. Substituting the state parameters of iron and basalt (iron: ρ_0_ = 7.680 g cm^−3^, c = 3.80 km s^−1^, and s = 1.58; ref. 2) and that of OC (ρ_0_ = 3.469 g cm^−3^, c = 3.7237 km s^−1^, and s = 1.2822; ref. 48) into these equations, we obtained *u*_(iron)_ = 0.30 km s^−1^, *v*_*i*(iron)_ = 1.18 km s^−1^, *U*_*s*(iron)_ = 4.28 km s^−1^ and *u*_(OC)_ = 0.64 km s^−1^, *v*_*i*(OC)_ = 1.51 km s^−1^, *U*_*s*(OC)_ = 4.54 km s^−1^.

### Estimate of the projectile diameter (*D*_*proj*_)

The shock duration can be used to derive an order-of-magnitude diameter of the projectile (*D*_*proj*_). The duration of the shock pressure (*t*_*d*_) can be calculated as the sum of the duration of compression of the collision material (*t*_*c*_ = *D*_*proj*_*/U*_*s*_) and the time needed for expansion of the shock material, *t*_*r*_ = *(ρ*_*0/*_*ρ)D*_*proj*_*/C*_*r*_:


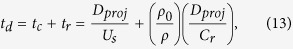


where *D*_*proj*_ is the diameter of the projectile, *ρ* is the density of the shocked material, and *C*_*r*_ is the rarefaction wave velocity. On the basis of a Murnaghan-type of equation of state^2^, *C*_*r*_is given by


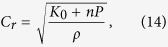


where *K*_*0*_ = *ρ*_*0*_*c*^2^ is the projectile’s bulk modulus and *n* = *4s* − *1* is a dimensionless constant. The density of the shocked material, *ρ*, can also be obtained as follows:


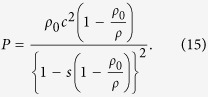


For NWA 8003, the shock pressure (*P* = 10 GPa) can be estimated from the high-pressure mineral assemblages. The solidification time (*t*_*s*_ = 72 ms) of 1-mm-wide shock-melt veins is the lower limit of the shock duration, *t*_*d*_, as discussed above. Substituting the state parameter, ρ_0_, and empirical constants (c and s) of basalt, iron, and OC^49^ into these equations, we obtained *ρ*_(basalt)_ = 3.65 g cm^−3^, *C*_*r*(basalt)_ = 4.50 km s^−1^, *D*_*proj*(basalt)_ = 0.17 km; *ρ*_(iron)_ = 8.27 g cm^−3^, *C*_*r*(iron)_ = 4.46 km s^−1^, *D*_*proj*(iron)_ = 0.16 km; and *ρ*_(OC)_ = 3.65 g cm^−3^, *C*_*r*(OC)_ = 4.71 km s^−1^, *D*_*proj*(OC)_ = 0.18 km.

### Estimating the crater diameter (*D*_*crater*_)

Using the impact velocity, *v*_*i*_, and the projectile size, *d*, calculated above, the crater diameter can be approximated using the pi-scaling relation^1^:


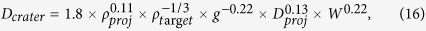


where *g* is the gravitational acceleration on Vesta, *g* = 0.22 ms^−2^ (refs 50, 51) and *W* is pure kinetic energy:


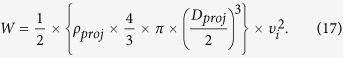


Substituting the values of the impact velocity and the projectile size for three types of projectiles into Equation (16) and equation (17), we obtained crater diameters created by projectiles composed of basalt, OC, and iron is 2.8 km, 2.9 km, and 3.2 km, respectively. Figure 6 shows that the crater diameter is a function of the shock pressure for impactors of various densities from 5 to 15 GPa assuming the same shock pulse of 72 ms.

## Additional Information

**How to cite this article**: Pang, R.-L. *et al.* High-pressure minerals in eucrite suggest a small source crater on Vesta. *Sci. Rep.* **6**, 26063; doi: 10.1038/srep26063 (2016).

## Supplementary Material

Supplementary Information

## References

[b1] Melosh H. J. Impact Cratering: A Geological Process. (Oxford Univ. Press, 2009).

[b2] Melosh H. J. The contact and compression stage of impact cratering. In Impact Cratering: processes and products. (eds Osinski G. R. & Pierazzo E.) 32–42 (Wiley-Blackwell, West Sussex, 2013).

[b3] Chen M., Sharp T. G., El Goresy A., Wopenka B. & Xie X. The majorite-pyrope + magnesiowüstite assemblage: constraints on the history of shock veins in chondrites. Science 271, 1570–1573 (1996).

[b4] Sharp T. G. & DeCarli P. S. Shock effects in meteorites. In Meteorites and the Early Solar System II. (eds Lauretta D. S. & McSween H. Y.) 653–677 (University of Arizona Press, 2006).

[b5] Gillet P., El Goresy A., Beck P. & Chen M. High pressure mineral assemblages in shocked meteorites and shocked terrestrial rocks: mechanisms of phase transformations and constraints to pressure and temperature histories. In *Advances in High-Pressure Mineralogy*. (ed Ohtani, E.) *Geol Soc Am Special paper* **421**, 57–82 (2007).

[b6] Gillet P. & El Goresy A. Shock events in the solar system: the message from minerals in terrestrial planets and asteroids. Annu. Rev. Earth Planet. Sci. 41, 12.1–12.29 (2013).

[b7] El Goresy A. *et al.* Shock-induced deformation of Shergottites: Shock-pressures and perturbations of magmatic ages on Mars. Geochim. Cosmochim. Acta 101, 233–262 (2013).

[b8] Kaneko S. *et al.* Discovery of stishovite in Apollo 15299 sample. Am. Mineral. 100, 1308–1311 (2015).

[b9] French B. M. & Koeberl C. The convincing identification of terrestrial meteorite impact structures: What works, what doesn’t, and why. Earth-Sci. Rev. 98, 123–170 (2010).

[b10] Langenhorst F. & Poirier J. P. Anatomy of black veins in Zagami: clues to the formation of high-pressure phases. Earth Planet. Sci. Lett. 184, 37–55 (2000).

[b11] Ohtani E. *et al.* Formation of high-pressure minerals in shocked L6 chondrite Yamato 791384: constraints on shock conditions and parent body size. Earth Planet. Sci. Lett. 227, 505–515 (2004).

[b12] Beck P., Gillet Ph., El Goresy A. & Mostefaoui S. Timescales of shock processes in chondritic and martian meteorites. Nature 435, 1071–1074 (2005). 15973403 10.1038/nature03616

[b13] Ozawa S. *et al.* Jadeite in Chelyabinsk meteorite and the nature of an impact event on its parent body. Sci. Rep. 4, 5033 (2014). 24852082 10.1038/srep05033 PMC4030444

[b14] Marchi S. *et al.* The violent collisional history of asteroid 4 Vesta. Science 336, 690–694 (2012). 22582255 10.1126/science.1218757

[b15] Schenk P. *et al.* The geologically recent giant impact basins at Vesta’s south pole. Science 336, 694–697 (2012). 22582256 10.1126/science.1223272

[b16] Migliorini F. *et al.* Vesta fragments from ν_6_ and 3:1 resonances: Implications for V-type near-Earth asteroids and howardite, eucrite and diogenite meteorites. Meteorit. Planet. Sci. 32, 903–916 (1997).

[b17] Melosh H. J. Impact ejection, spallation, and the origin of meteorites. Icarus 59, 234–260 (1984).

[b18] Miyahara M. *et al.* Discovery of coesite and stishovite in eucrite. Proc. Natl Acad. Sci. USA 111, 10939–10942 (2014). 25028493 10.1073/pnas.1404247111 PMC4121824

[b19] Zhang A. C. *et al.* Petrogenesis of lunar meteorite Northwest Africa 2977: constraints from *in situ* microprobe results. Meteorit. Planet. Sci. 45, 1929–1947 (2010).

[b20] Zhang A. C. *et al.* Impact melting of lunar meteorite Dhofar 458: Evidence from polycrystalline texture and decomposition of zircon. Meteorit. Planet. Sci. 46, 103–115 (2011).

[b21] Ohtani E. *et al.* Coesite and stishovite in a shocked lunar meteorite, Asuka-881757, and impact events in lunar surface. Proc. Natl Acad. Sci. USA 108, 463–466 (2011). 21187434 10.1073/pnas.1009338108 PMC3021006

[b22] Miyahara M. *et al.* Discovery of seifertite in a shocked lunar meteorite. Nat. Commun. 4, 1737, http://dx.doi.org/10.1038/ncomms2733 (2013). 23612278 10.1038/ncomms2733

[b23] Lorenz K. A., Nazarov M. A., Kurat G., Brandstätter F. & Ntaflos T. Foreign meteoritic material of howardites and polymict eucrites. Petrologiya 15, 115–132 (2007).

[b24] Ryder G., Norman M. D. & Score R. A. The distinction of pristine from meteorite-contaminated highlands rocks using metal compositions. Proc. Lunar Planet. Sci. Conf. 11, 471–479 (1980).

[b25] Brey G. P. & Köhler T. Geothermobarometry in four-phase lherzolites II. New thermobarometers, and practical assessment of existing thermobarometers. J. Petrol. 31, 1353–1378 (1990).

[b26] Walton E. L., Sharp T. G., Hu J. & Filiberto J. Heterogeneous mineral assemblages in martian meteorite Tissint as a result of a recent small impact event on Mars. Geochim. Cosmochim. Acta 140, 334–348 (2014).

[b27] Ma C. *et al.* Tissintite, (Ca,Na,□)AlSi_2_O_6_, a highly-defective, shock-induced, high-pressure clinopyroxene in the Tissint martian meteorite. Earth Planet. Sci. Lett. 422, 194–205 (2015).

[b28] Sharp T. G., Walton E. L. & Hu J. Shock effects in NWA 8159: Evidence for a modest shock pressure and a large impacting body. Lunar Planet. Sci. Conf. 46, #1939 (2015).

[b29] Wenk H. R. & Weiss L. E. Al-rich calcic pyroxene in pseudotachiylite: An indicator of high pressure and high temperature? Tectonophysics 84, 329–341 (1982).

[b30] Zhao S., Nee P., Green H. W. & Dobrzhinetskaya L. F. Ca-Eskola component in clinopyroxene: Experimental studies at high pressures and high temperatures in multianvil apparatus. Earth Planet. Sci. Lett. 307, 517–524 (2011).

[b31] Bläß U. W. Shock-induced formation mechanism of seifertite in shergottites. Phys. Chem. Mineral. 40, 425–437 (2013).

[b32] He Q. *et al.* Petrography and geochemistry of the enriched basaltic shergottite Northwest Africa 2975. Meteorit. Planet. Sci. 50, 2024–2044 (2015).

[b33] Kubo T., Kato T., Higo Y. & Funakoshi K. I. Curious kinetic behavior in silica polymorphs solves seifertite puzzle in shocked meteorite. Sci. Adv. 1, e1500075 (2015). 26601182 10.1126/sciadv.1500075 PMC4640644

[b34] Perrillat J. P., Daniel I., Lardeaux J. M. & Cardon H. Kinetics of the coesite-quartz transition: Application to the exhumation of ultrahigh-pressure rocks. J. Petrol. 44, 773–788 (2003).

[b35] Hemley R. J., Jephcoat A. P., Mao H. K., Ming L. C. & Manghnani M. H. Pressure-induced amorphization of crystalline silica. Nature 334, 52–54 (1988).

[b36] Dubrovinsky L. S. *et al.* Pressure-induced transformations of cristobalite. Chem. Phys. Lett. 333, 264–270 (2001).

[b37] Liu L. Phase relations in the system diopside-jadeite at high pressures and high temperatures. Earth Planet. Sci. Lett. 47, 398–402 (1980).

[b38] Gleason A. E. *et al.* Ultrafast visualization of crystallization and grain growth in shock-compressed SiO_2_. Nat. Commun. 6, 819 (2015). 10.1038/ncomms9191 PMC4569796 26337754

[b39] Presnall D. C. Phase diagrams of Earth-forming minerals. In Mineral Physics & Crystallography, A Handbook of Physical Constants, (ed Ahrens T. J.) 248–268 (American Geophysical Union, Washington, DC, 1995).

[b40] Yasuda A., Fujii T. & Kurita K. Melting phase relations of an anhydrous mid-ocean ridge basalt from 3 to 20 GPa: implications for the behavior of subducted oceanic crust in the mantle. J Geophys. Res. 99, 9401–9414 (1994).

[b41] Aoki I. & Takahashi E. Density of MORB eclogite in the upper mantle. Phys. Earth Planet. Inter. 143–144, 129–143 (2004).

[b42] Turcotte D. L. & Schubert G. Geodynamics: Second Edition. (Cambridge University Press, 2002).

[b43] Bottke W. F., Nolan M. C., Greenberg R. & Kolvoord R. A. Velocity distributions among colliding asteroids. Icarus 107, 255–268 (1994).

[b44] Marchi S. *et al.* High-velocity collisions from the lunar cataclysm recorded in asteroidal meteorites. Nat. Geosci. 6, 303–307 (2013).

[b45] Baziotis I. P. *et al.* The Tissint Martian meteorite as evidence for the largest impact excavation. Nat. Commun. 4, 1404 (2013). 23360995 10.1038/ncomms2414

[b46] Turcotte D. L. & Schubert G. Geodynamics (J. Wiley and Sons, 1982).

[b47] Stöffler, D. & Grieve, R. A. F. In *Metamorphic rocks*: *A classification and glossary of terms, recommendations of the International Union of Geological Sciences*. (eds Fettes, D. & Desmons, J.) 82–92, 111–125, and 126–242 (Cambridge Univ. Press, 2007).

[b48] Dai C., Jin X., Fu S., Shi S. & Wang D. The equation-of-states of Jilin ordinary chondrite and Nandan iron meteorite. Sci. in China (Series D) 40, 403–410 (1997).

[b49] van Thiel M. Compendium of shock wave data, University of California, Lawrence Radiation Laboratory, UCRL-50801, Vol. 1, Rev. 1, pp 9 (1977).

[b50] Reddy V. *et al.* Delivery of dark material to Vesta via carbonaceous chondritic impacts. Icarus 221, 544–559 (2012).

[b51] Ermakov A. I. *et al.* Constraints on Vesta’s interior structure using gravity and shape models from the Dawn mission. Icarus 240, 146–160 (2014).

