# Supplementary material for: High-pressure minerals in eucrite suggest a small source crater on Vesta
Source: Sci Rep. 2016 May 16;6:26063. doi: 10.1038/srep26063 (PMC4867502; doi:10.1038/srep26063)
Supplement: Supplementary Information [file srep26063-s1.pdf]

High-pressure minerals in eucrite suggest a small source crater on Vesta

Run-Lian Pang[1], Ai-Cheng Zhang[1,*], Shu-Zhou Wang[1], Ru-Cheng Wang[1] & Hisayoshi Yurimoto[2]

[1] State Key Laboratory for Mineral Deposits Research, School of Earth Sciences and Engineering, Nanjing University, Nanjing 210046, China.

[2] Department of Natural History Sciences, Hokkaido University, Sapporo 060-0810, Japan.

*Correspondence and requests for materials should be addressed to A.C.Z (e-mail: aczhang@nju.edu.cn)

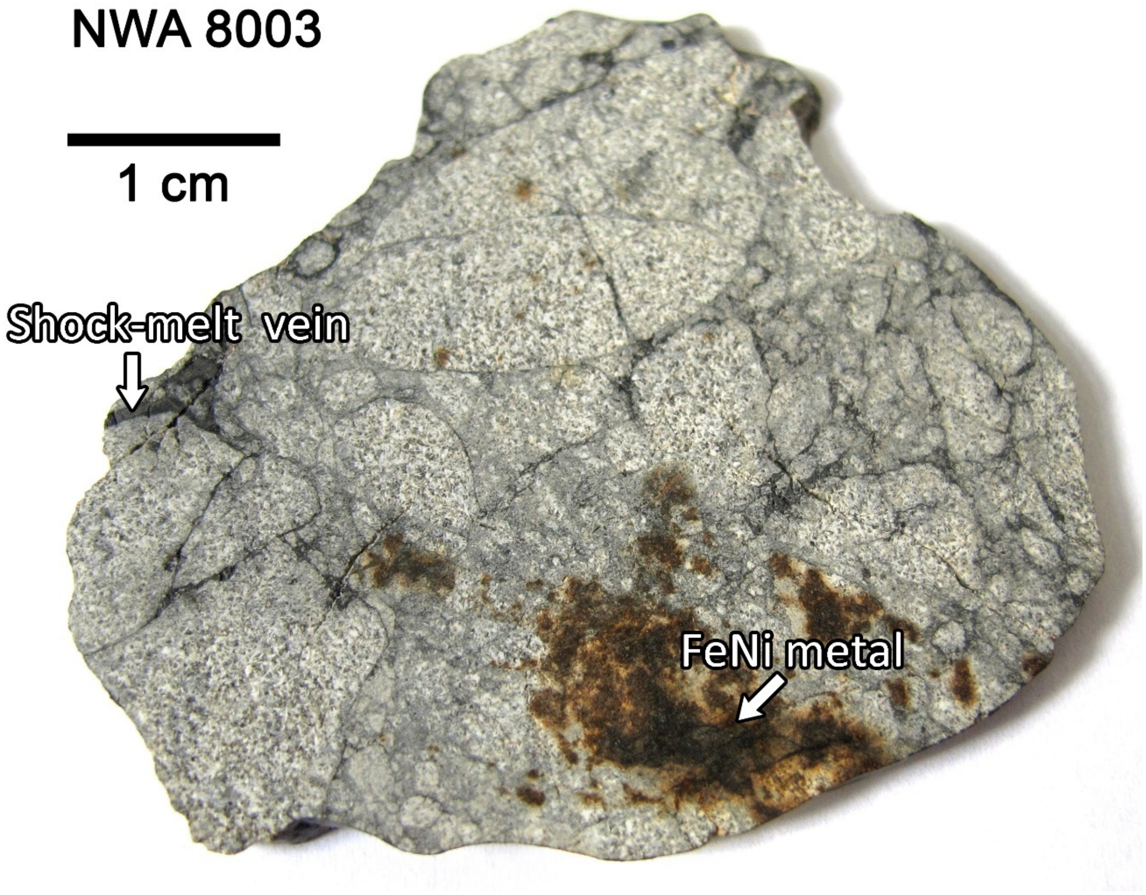

Supplementary Figure 1. Photograph of a fragment of a shocked eucrite NWA 8003. In the fragment, the FeNi metal grains have been partially altered due to terrestrial weathering. The width of the veins is up to ~1 mm.

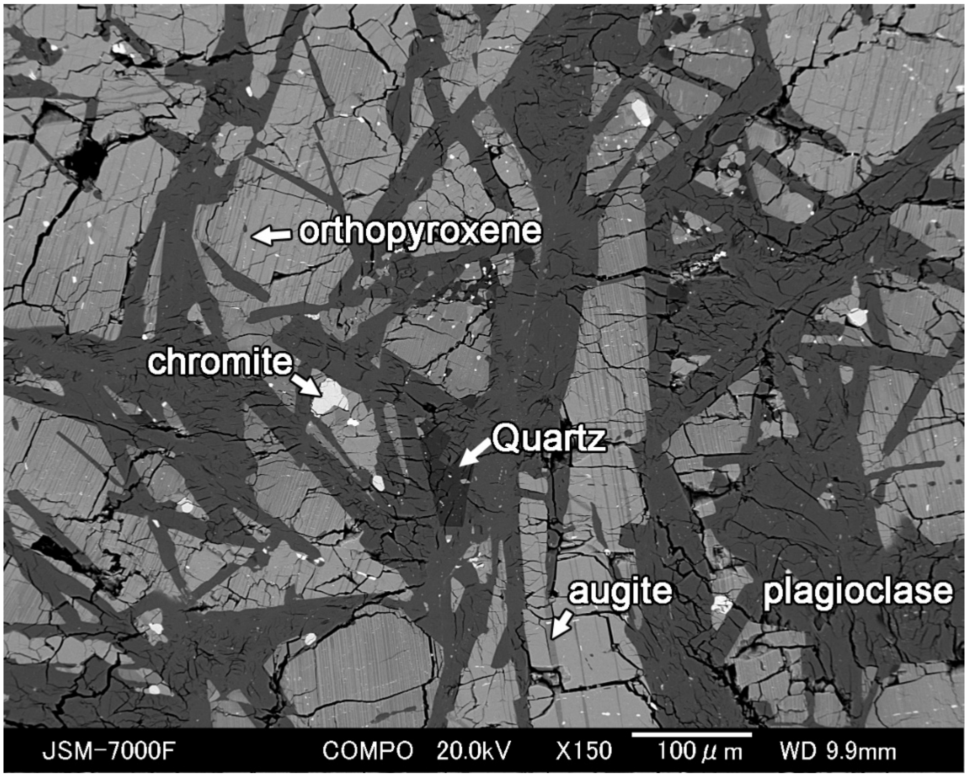

Supplementary Figure 2. Backscattered electron image of texture of the host rock of NWA 8003. Pyroxene grains show thin exsolution lamellae of augite within orthopyroxene. Lath-like plagioclase crystals contain abundant irregular fractures.

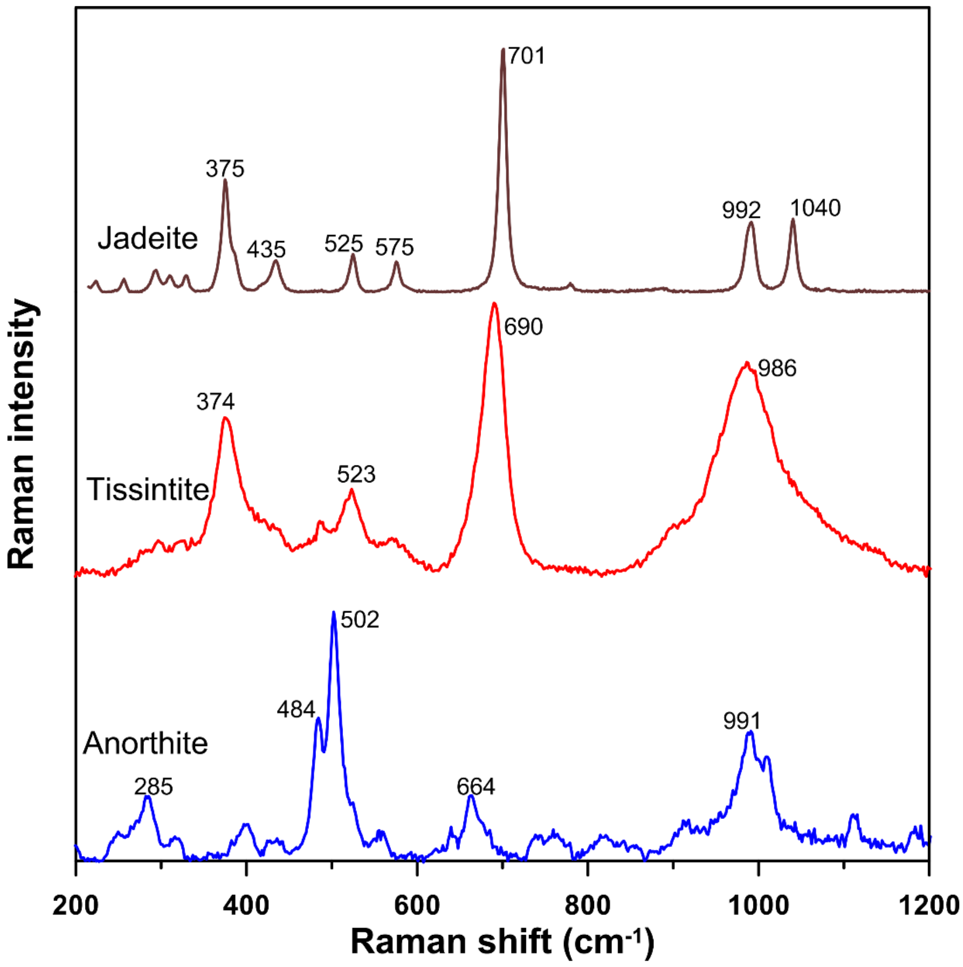

Supplementary Figure 3. Raman spectra of anorthite, tissintite in NWA 8003 and jadeite from San Benito County, California, USA (http://Ruff.info/Jadeite/R050220).

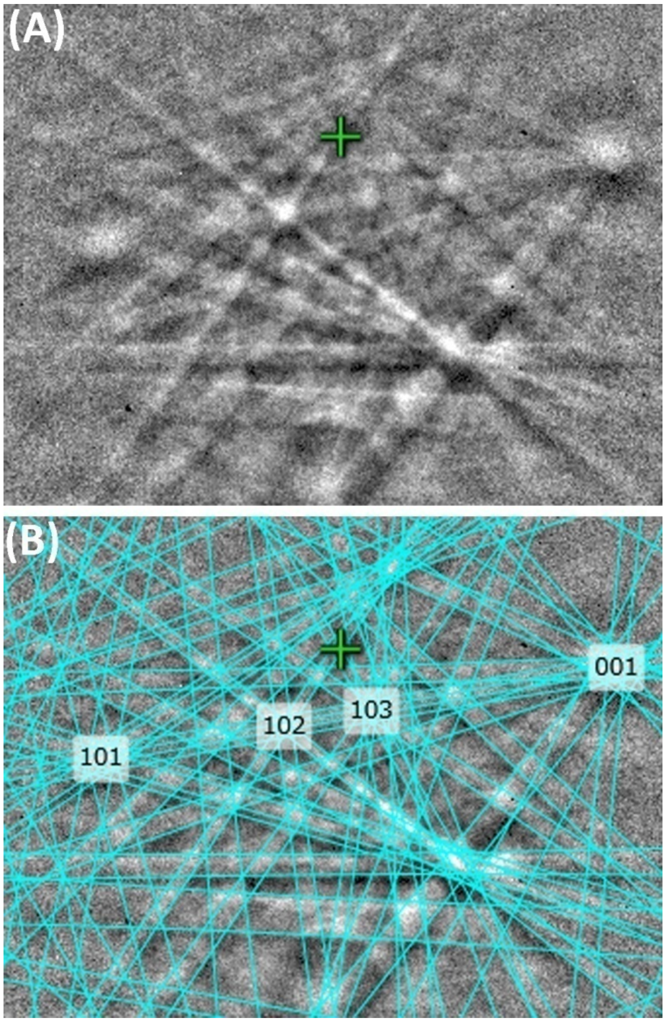

Supplementary Figure 4. EBSD pattern (A) of tissintite in NWA 8003 and the pattern (B) indexed with the *C2/c* clinopyroxene structure (MAD=0.30).

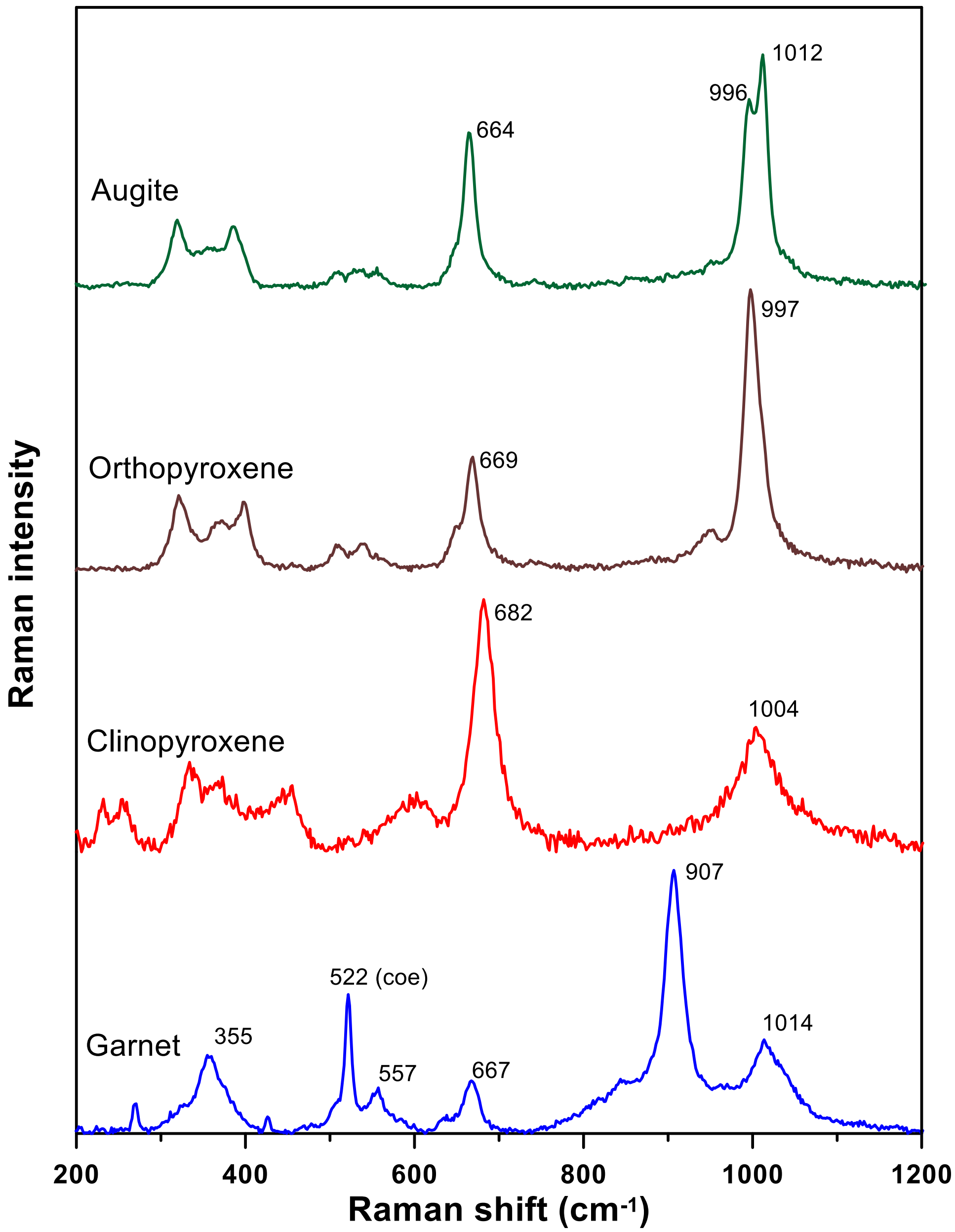

Supplementary Figure 5. Raman spectra of augite and orthopyroxene from the host rock and clinopyroxene and garnet in melt veins of NWA 8003.

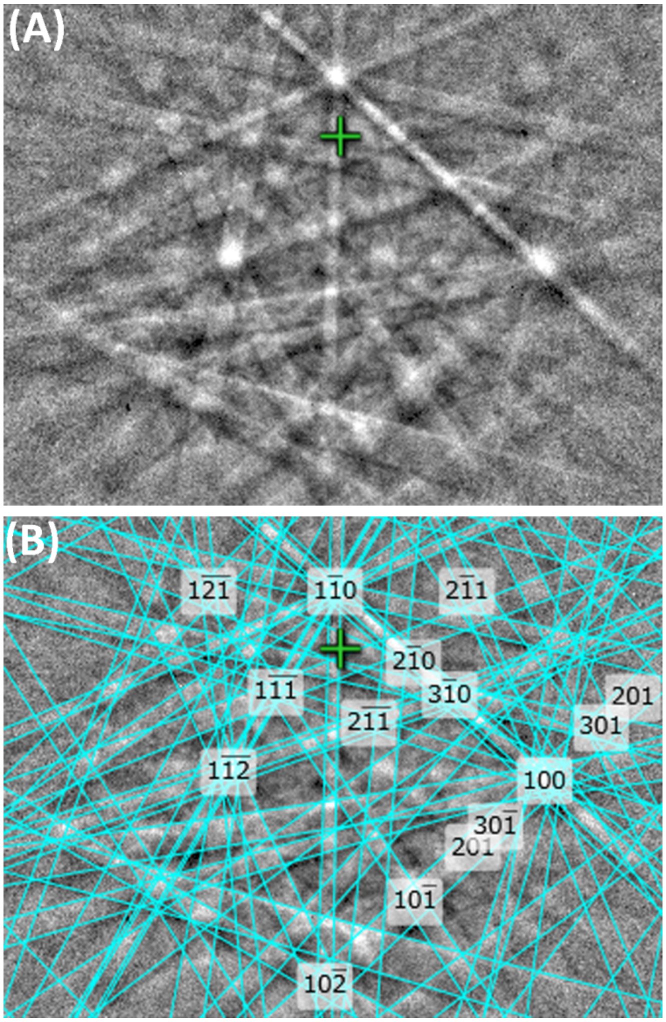

Supplementary Figure 6. EBSD pattern (A) of clinopyroxene in melt veins from NWA 8003 and the pattern (B) indexed with the *C2/c* clinopyroxene structure (MAD=0.25).

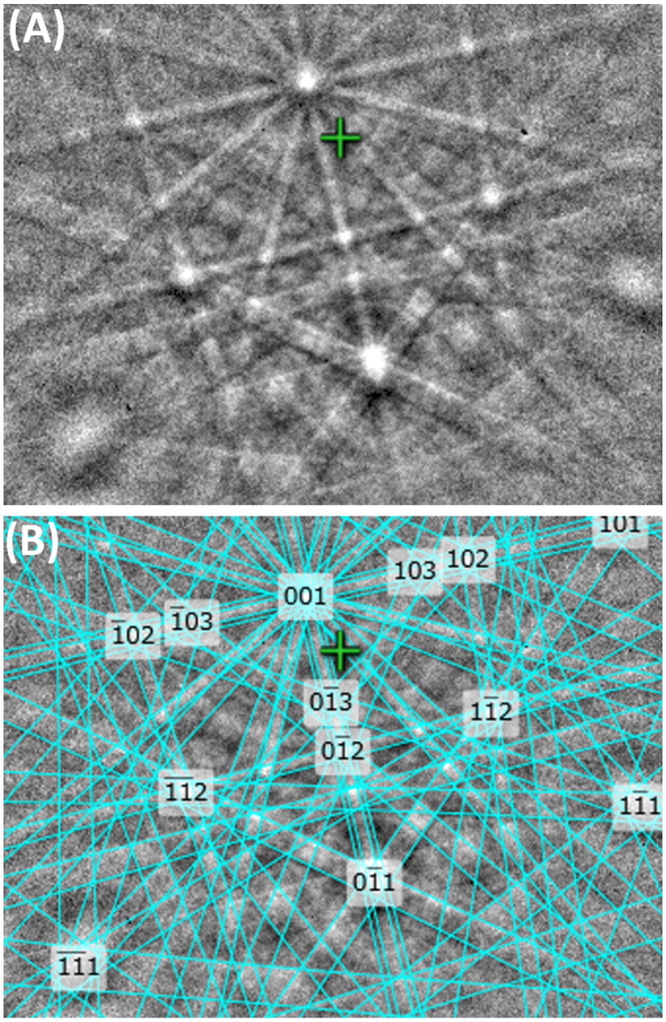

Supplementary Figure 7. EBSD pattern (A) of garnet in NWA 8003 and the pattern (B) indexed with the isometric *Ia-3d* garnet structure (MAD=0.22).

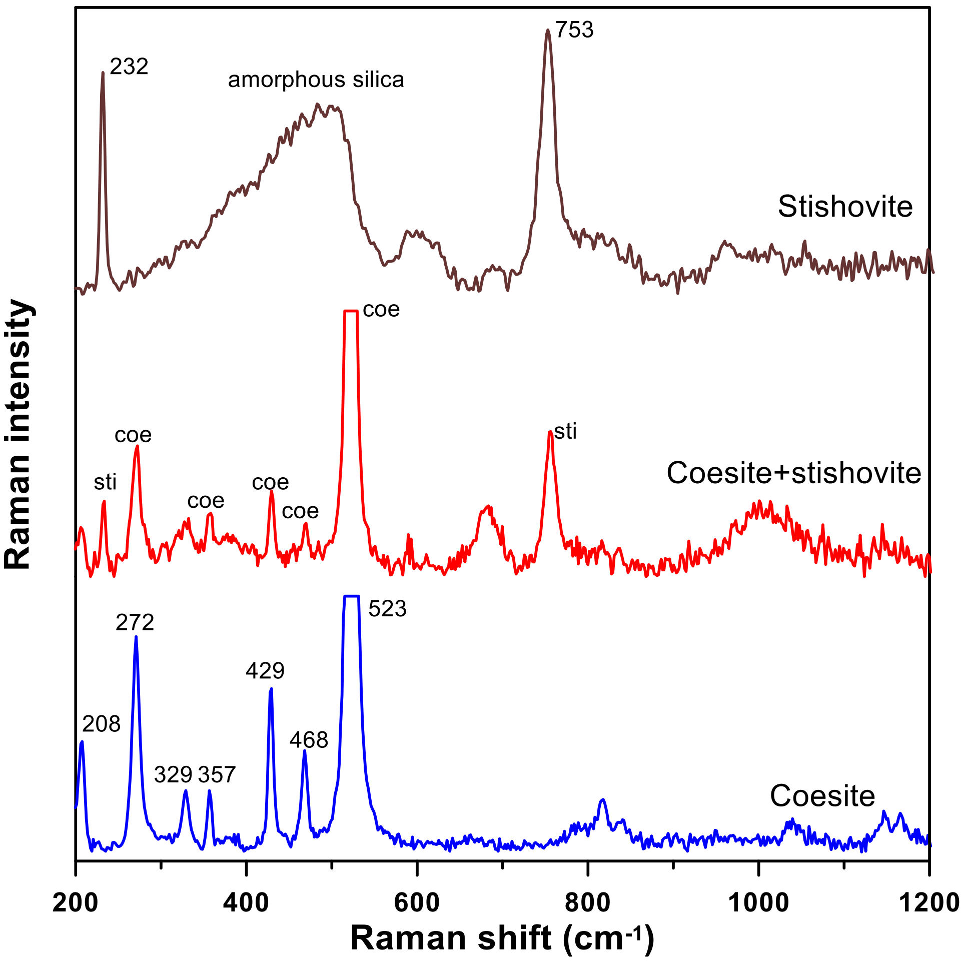

Supplementary Figure 8. Raman spectra of high-pressure polymorphs of silica in NWA 8003.

Supplementary Table 1. EPMA compositions of ferromagnesian pyroxene (wt%) in NWA 8003.

	Orthopyroxene in the host rock			Augite in the host rock			Clinopyroxene in clinopyroxene-dominant zone/vein			Clinopyroxene in eclogitic mineral assemblage		
	Range	Average (n=12)	*Stdev*	Range	Average (n=14)	*Stdev*	Range	Average (n=19)	*Stdev*	Range	Average (n=13)	*Stdev*
SiO_2	49.1-49.9	49.5	*0.3*	50.9-51.8	51.3	*0.2*	46.2-51.7	48.9	*1.3*	47.7-55.7	52.0	*2.3*
TiO_2	0.06-0.43	0.13	*0.10*	0.14-0.31	0.22	*0.05*	0.19-1.13	0.65	*0.30*	0.19-0.85	0.47	*0.21*
Al_2O_3	0.06-0.41	0.14	*0.09*	0.30-0.56	0.43	*0.06*	15.0-24.4	17.7	*2.1*	14.3-26.8	17.4	*3.6*
Cr_2O_3	0-0.37	0.07	*0.10*	0.06-0.22	0.13	*0.04*	0.05-0.27	0.17	*0.08*	0.05-0.22	0.16	*0.06*
MgO	10.4-11.1	10.7	*0.2*	9.15-9.61	9.34	*0.14*	2.41-6.56	4.54	*0.83*	2.07-6.03	4.62	*1.04*
FeO	37.0-37.8	37.4	*0.3*	16.9-17.9	17.4	*0.3*	11.0-20. 9	15.2	*2.6*	5.81-14.1	11.4	*2.3*
MnO	1.09-1.23	1.15	*0.05*	0.48-0.59	0.52	*0.03*	0.21-0.58	0.36	*0.11*	0.10-0.52	0.32	*0.13*
CaO	0.75-1.65	1.02	*0.25*	20.3-20.9	20.7	*0.2*	10.8-13.2	11.9	*0.7*	12.0-16.7	13.0	*1.3*
Na_2O	0-0.04	0.02	*0.02*	0.04-0.10	0.07	*0.02*	0.42-1.02	0.69	*0.14*	0.77-1.43	1.13	*0.22*
K_2O	bd	bd	*bd*	bd	bd	*bd*	0-0.10	0.05	*0.03*	0.03-0.15	0.07	*0.03*
Total	99.30-100.6	100.1		99.47-100.9	100.4		99.35-100.7	100.0		100.1-101.0	100.6	
							Based on 6 oxygen atoms					
Si	1.981-2.003	1.995	*0.006*	1.976-1.993	1.985	*0.005*	1.707-1.885	1.796	*0.040*	1.681-1.980	1.866	*0.083*
Ti	0.002-0.013	0.004	*0.003*	0.004-0.009	0.006	*0.001*	0.005-0.032	0.018	*0.008*	0.005-0.023	0.013	*0.006*
Al	0.004-0.020	0.007	*0.004*	0.014-0.025	0.020	*0.003*	0.657-1.024	0.763	*0.083*	0.602-1.110	0.732	*0.146*
Cr	0-0.012	0.002	*0.003*	0.002-0.007	0.004	*0.001*	0.001-0.008	0.005	*0.002*	0.001-0.006	0.005	*0.002*
Mg	0.6340.672	0.648	*0.012*	0.532-0.553	0.542	*0.007*	0.129-0.361	0.250	*0.046*	0.109-0.329	0.249	*0.058*
Fe	1.234-1.278	1.258	*0.011*	0.547-0.577	0.561	*0.008*	0.326-0.659	0.466	*0.087*	0.171-0.427	0.340	*0.071*
Mn	0.037-0.042	0.039	*0.002*	0.016-0.019	0.017	*0.001*	0.006-0.018	0.011	*0.004*	0.003-0.016	0.010	*0.004*
Ca	0.033-0.071	0.044	*0.011*	0.843-0.870	0.858	*0.008*	0.426-0.507	0.467	*0.024*	0.456-0.629	0.498	*0.046*
Na	0-0.003	0.001	*0.001*	0.003-0.008	0.005	*0.002*	0.030-0.071	0.049	*0.010*	0.054-0.099	0.078	*0.015*
K	bd	bd	*bd*	bd	bd	*bd*	0-0.005	0.002	*0.001*	0.001-0.007	0.003	*0.001*
ΣCations	3.990-4.007	3.998		3.994-4.010	3.999		3.767-3.888	3.828		3.736-3.860	3.794	
En	32.6-34.0	33.2	*0.5*	27.3-28.3	27.6	*0.3*						
Fs	63.4-65.6	64.5	*0.6*	28.0-29.1	28.6	*0.3*						
Wo	1.7-3.7	2.3	*0.5*	42.8-44.6	43.8	*0.4*						
Ca-Esk							22-47	34	*7*	28-53	41	*8*

bd: Below detection limit.

Ca-Esk = 100*(Al$_{tot}$-2*(2-Si-Ti)-Na) in mole.

Supplementary Table 2. EPMA compositions of plagioclase, tissintite, and maskelynite (wt%) in NWA 8003.

	Plagioclase			Tissintite			Maskelynite		
	range	average	*stdev*	range	average	*stdev*	range	average	*stdev*
SiO_2	45.3-47.2	46.3	*0.8*	45.6-49.3	47.1	*1.1*	45.2-47.0	46.2	*0.7*
Al_2O_3	33.8-34.8	34.2	*0.4*	32.4-35.7	34.1	*0.7*	33.1-34.7	34.2	*0.5*
FeO	0.13-0.46	0.25	*0.15*	0.19-0.70	0.40	*0.16*	0.14-0.73	0.31	*0.17*
CaO	17.0-18.3	17.6	*0.7*	16.0-18.4	17.3	*0.7*	17.2-18.5	17.8	*0.5*
Na_2O	1.00-1.75	1.38	*0.35*	1.01-1.89	1.32	*0.24*	0.81-1.72	1.28	*0.32*
K_2O	0.03-0.08	0.06	*0.02*	0.04-0.08	0.05	*0.01*	0.04-0.09	0.06	*0.02*
Total	99.5-100.3	99.89		99.31-100.9	100.3		98.42-100.5	99.85	
	Based on 8 oxygen atoms			*Based on 6 oxygen atoms*					
Si	2.096-2.161	2.136	*0.031*	1.574-1.687	1.618	*0.031*			
Al	1.836-1.895	1.856	*0.027*	1.304-1.444	1.380	*0.032*			
Fe	0.005-0.018	0.009	*0.006*	0.005-0.020	0.012	*0.005*			
Ca	0.834-0.910	0.869	*0.036*	0.584-0.683	0.636	*0.029*			
Na	0.090-0.155	0.123	*0.030*	0.067-0.125	0.087	*0.016*			
K	0.002-0.005	0.004	*0.001*	0.002-0.003	0.002	*0.001*			
ΣCations	4.994-5.002	4.999		3.703-3.754	3.737				
An	83.9-90.8	87.3	*3.2*						
Ab	9.0-15.6	12.4	*3.1*						
Or	0.2-0.5	0.3	*0.1*						

Supplementary Table 3. Representative bulk compositions (wt%) of melt veins in NWA 8003*

	Wide veins with garnet								Thin veins	
SiO_2	50.5	50.7	51.3	50.2	50.5	50.7	50.1	49.9	49.3	49.8
TiO_2	0.99	1.10	1.03	0.89	0.92	1.02	0.93	0.90	0.89	0.96
Al_2O_3	15.5	15.3	14.9	15.8	15.8	15.8	15.9	15.5	16.2	16.2
MgO	5.27	5.02	4.93	5.32	5.14	5.14	5.27	5.47	5.29	5.35
FeO	16.1	16.4	16.3	16.2	16.0	15.6	15.9	16.4	16.0	16.0
CaO	10.6	10.5	10.6	10.7	10.8	10.8	11.0	10.9	11.3	10.9
Na_2O	0.99	0.92	1.02	0.96	0.94	0.93	0.95	0.99	1.09	0.96
Total	100	100	100	100	100	100	100	100	100	100

*The bulk compositions of melt veins are determined with an energy-dispersive spectrometer (EDS) installed on a scanning electron microscope (JEOL 6490). The EDS data were collected by using Oxford INCA software.

Supplementary Table 4. EPMA compositions of garnet (wt%) in NWA 8003.

	Eclogitic mineral assemblage			*Garnet + glass zone*		
	Range	average	*Stdev (n=12)*	Range	average	*Stdev (n=9)*
SiO_2	40.5-41.6	41.0	*0.3*	40.1-41.0	40.6	*0.3*
TiO_2	0.37-0.77	0.48	*0.10*	0.39-0.78	0.52	*0.14*
Al_2O_3	19.2-20.2	19.7	*0.4*	19.9-20.6	20.2	*0.2*
Cr_2O_3	0.07-0.41	0.22	*0.12*	0.30-0.42	0.35	*0.04*
MgO	6.62-7.41	6.99	*0.22*	6.94-7.30	7.10	*0.12*
FeO	18.5-20.7	19.2	*0.6*	18.3-19.2	18.9	*0.3*
MnO	0.37-0.51	0.43	*0.04*	0.37-0.48	0.44	*0.04*
CaO	11.6-12.8	12.3	*0.3*	12.0-12.5	12.3	*0.2*
Na_2O	0.30-0.53	0.38	*0.07*	0.25-0.42	0.33	*0.06*
Total	99.36-101.8	100.7		100.0-101.6	100.8	
			Based on 12 oxygen atoms			
Si	3.091-3.134	3.109	*0.012*	3.0653.092	3.077	*0.010*
Ti	0.021-0.044	0.027	*0.006*	0.022-0.044	0.029	*0.008*
Al	1.702-1.802	1.763	*0.030*	1.771-1.831	1.803	*0.023*
Cr	0.004-0.025	0.013	*0.007*	0.018-0.025	0.021	*0.002*
Mg	0.748-0.841	0.796	*0.025*	0.792-0.826	0.807	*0.012*
Fe	1.162-1.318	1.213	*0.040*	1.153-1.209	1.194	*0.019*
Mn	0.023-0.033	0.027	*0.002*	0.024-0.030	0.028	*0.003*
Ca	0.951-1.038	0.998	*0.023*	0.982-1.009	0.999	*0.009*
Na	0.044-0.078	0.055	*0.010*	0.037-0.061	0.048	*0.008*
ΣCations	7.991-8.012	8.003		8.003-8.009	8.006	

bd: Below detection limit.
